# Skin Involvement of Idiopathic Granulomatous Mastitis: Sonographic, Clinical, and Histopathological Features

**DOI:** 10.1155/tbj/7224219

**Published:** 2025-09-24

**Authors:** Fatih Işık, Erdal Pala, Fatih Alper, Sevilay Ozmen, Elif Demirci, Hasan Abbasguliyev, Müfide Nuran Akçay

**Affiliations:** ^1^Department of Radiology, Erzurum Training and Research Hospital, Erzurum, Turkey; ^2^Department of Dermatology, Atatürk University Faculty of Medicine, Erzurum, Turkey; ^3^Department of Radiology, Atatürk University Faculty of Medicine, Erzurum, Turkey; ^4^Department of Pathology, Atatürk University Faculty of Medicine, Erzurum, Turkey; ^5^Department of General Surgery, Atatürk University Faculty of Medicine, Erzurum, Turkey

**Keywords:** breast inflammation, idiopathic granulomatous mastitis (IGM), skin involvement, sonographic and histopathological features

## Abstract

**Objectives:** Idiopathic granulomatous mastitis (IGM) is a rare benign breast disease in which cutaneous involvement is insufficiently characterized. This study aimed to evaluate the sonographic, clinical, and histopathological features of skin manifestations in IGM.

**Methods:** We retrospectively analyzed 138 women with biopsy-proven IGM who underwent breast and skin ultrasonography between 2023 and 2024. Clinical cutaneous findings were documented, and 14 patients with visible skin lesions underwent additional punch biopsy for histopathological evaluation. Sonographic and clinical features were stratified according to symptom duration (0–3, 4–6, 7–9, and ≥ 10 weeks).

**Results:** Cutaneous lesions were identified in 84/138 patients (60.9%). Sonographic findings followed a sequential distribution: fibrous echogenicity loss in early disease (11/14, 78.6%), vacuolar structures at 4–6 weeks (11/19, 57.9%), dermo-subcutaneous blurring at 7–9 weeks (9/34, 26.5%), and advanced features such as fistula formation (11/17, 64.7%) and dermo-subcutaneous disruption (11/17, 64.7%) beyond 10 weeks. Clinical findings paralleled imaging, with erythema and papulopustular lesions predominating early (13/14, 92.9%), erythema nodosum peaking at 4–6 weeks (7/19, 36.8%), and ulceration and fistula formation emerging after ≥ 7 weeks (11/17, 64.7% at ≥ 10 weeks). Histopathological analysis of 14 skin biopsies demonstrated nonspecific inflammatory changes without granuloma formation.

**Conclusions:** IGM demonstrates sequential sonographic and clinical cutaneous patterns associated with symptom duration. Early erythematous and papulopustular changes progress to ulceration and fistula formation in prolonged disease. Although supportive in suggesting cutaneous involvement, these features are not diagnostic, and histopathological confirmation remains essential. Prospective studies are warranted to further define the clinical and histological course of cutaneous changes in IGM.

## 1. Introduction

Idiopathic granulomatous mastitis (IGM) is a rare, benign inflammatory breast disease that predominantly affects women of reproductive age, particularly among Latin and Asian populations [1, 2]. First described by Kessler and Wolloch in 1972 [3], IGM has been associated with a variety of contributing factors, including hormonal changes such as pregnancy, breastfeeding, and oral contraceptive use [4]; autoimmune disorders [5, 6]; diabetes mellitus [7]; and genetic predispositions [8–10].

Despite its increasing clinical recognition, the imaging characteristics of IGM remain underreported in the radiology literature, with the existing publications primarily emphasizing sonographic abnormalities in younger patients. Clinically, IGM may present with localized symptoms such as fistulas, abscesses, mass-like lesions, nipple retraction, ulceration, and skin thickening. The etiology of IGM is not yet fully understood, although it is presumed to involve an abnormal immune response triggered by infection or trauma [1]. Its responsiveness to corticosteroids and immunosuppressive agents such as methotrexate supports an autoimmune pathogenesis [10].

Although skin involvement is a common clinical feature of IGM, no dedicated studies have specifically investigated the imaging or histopathological characteristics of this involvement. This study aims to bridge this gap by presenting novel findings related to the sonographic, clinical, and histological features of skin involvement in IGM.

### 1.1. Demographics

Granulomatous mastitis (GM) typically affects women of reproductive age, which may skew the perception of its true demographic distribution [11, 12]. While the exact incidence of IGM is unknown, the U.S. Centers for Disease Control estimated an annual prevalence of 2.4 per 100,000 women in Indianapolis from 2006 to 2008 [11, 13]. In a systematic review by Martinez-Ramos et al., Turkey was reported as the leading country in terms of the number of IGM-related publications [14].

In our institution, anecdotal evidence suggests that Turkish women, followed by those of Caucasian ethnicity, represent the majority of cases. All patients who received intralesional steroid injections for biopsy-confirmed IGM between 2023 and 2024 had a documented history of breastfeeding for at least 1-2 years before the onset of symptoms. Notably, the nonlactating breast was typically the most affected side.

### 1.2. Clinical Manifestations

Another common feature is the development of uni- or multifocal abscesses as the disease progresses. Prior studies have demonstrated abscess formation in various patient cohorts, particularly in regions with higher disease prevalence [15], histopathologic series [11], and cohorts treated surgically or medically [16, 17].

## 2. Materials and Methods

### 2.1. Patient Selection

Since 2016, patients with histopathologically confirmed IGM at our center have been treated with peri-intralesional steroid injections. For this study, sonographic evaluations of the breast parenchyma and overlying skin were performed in 138 patients diagnosed with IGM who presented for intralesional steroid therapy between 2023 and 2024. Upon clinical examination, 84 of these patients exhibited cutaneous lesions. Of those, 14 patients who provided informed consent underwent punch biopsy of the breast skin, which was performed in collaboration with the dermatology department.

### 2.2. Inclusion and Exclusion Criteria

#### 2.2.1. Inclusion Criteria

• The inclusion criteria include histopathologically confirmed diagnosis of IGM (all included patients had definitive biopsy-proven IGM). Among these, 14 patients who presented with clinically visible cutaneous lesions and consented to undergo punch biopsy of the breast skin were additionally included for histopathological evaluation of skin involvement.

#### 2.2.2. Exclusion Criteria

The exclusion criteria include the following:

• Histopathological findings inconsistent with GM
• Evidence of tuberculosis or other granulomatous diseases based on histopathology or laboratory data
• Clinical history suggestive of prior tuberculosis or another granulomatous disease
• Presence of infectious skin lesions that showed clinical or microbiological response to antibiotic therapy (e.g., Corynebacterium-associated lesions)

Patients with infectious mastitis were excluded based on clinical findings, negative microbiological stains (Ziehl–Neelsen, PAS, and GMS), and culture results when available, thereby ensuring that bacterial and fungal infections were ruled out prior to confirming the diagnosis of IGM.

### 2.3. Ultrasound Protocol

All sonographic examinations were performed using a Philips PureWave eL18-4 linear-array transducer with a frequency range of 2–22 MHz. Patients were positioned in the supine or supine oblique position with the ipsilateral arm elevated to allow optimal exposure of the breast and axillary regions. Both breasts and the overlying skin were systematically evaluated in transverse and longitudinal planes.

High-frequency imaging was utilized to assess superficial structures, including the dermis and subcutaneous tissue, while lower frequencies allowed deeper tissue penetration when necessary. Special attention was given to identifying cutaneous thickening, sinus tract formation, abscess cavities, hypoechoic masses, and echogenic changes in the skin layers suggestive of granulomatous inflammation. The sonographic findings were documented and archived for subsequent review and correlation with clinical and histopathological data.

### 2.4. Biopsy Procedures

All breast core needle biopsies were performed in our institution by radiologists in the breast imaging unit, ensuring histopathological confirmation of IGM. No patients were referred with pre-existing pathology reports from other centers. In patients who had clinically visible cutaneous lesions and provided informed consent, a skin punch biopsy was scheduled for the following day. Thus, breast and skin biopsies were not strictly simultaneous but were performed in close temporal proximity when clinically indicated.

### 2.5. Histopathological Analysis

All punch biopsies of the breast skin were performed under sterile conditions by a dermatology specialist using a 4-mm punch tool. The samples were immediately fixed in 10% neutral buffered formalin and submitted to the pathology laboratory for routine processing.

Histological sections were stained with hematoxylin and eosin (H&E) and examined by two experienced pathologists blinded to the clinical and radiologic data. The primary histological features assessed included granulomatous inflammation, the presence of multinucleated giant cells, caseating or noncaseating necrosis, lymphoplasmacytic infiltration, and dermal involvement. Ziehl–Neelsen staining was used to exclude acid-fast bacilli, and periodic acid–Schiff (PAS) and Grocott's methenamine silver (GMS) stains were performed in cases where fungal infection was suspected. Cases that did not meet histopathological criteria for GM or showed findings suggestive of infectious etiology were excluded.

## 3. Results

### 3.1. Sonographic Findings

Between 2023 and 2024, breast and skin ultrasonography was performed in 138 patients with histopathologically confirmed IGM who presented for peri-intralesional steroid therapy. Of these, 84 patients exhibited visible skin lesions on clinical inspection. Among them, 14 patients provided informed consent and underwent punch biopsy of the skin.

Ultrasonography, the primary imaging modality in patients presenting with mastitis symptoms, was used to evaluate both breast parenchyma and skin layers. In 84 patients with cutaneous involvement, serial sonographic examinations revealed a progressive alteration in cutaneous architecture over time.

**Figure 1:**
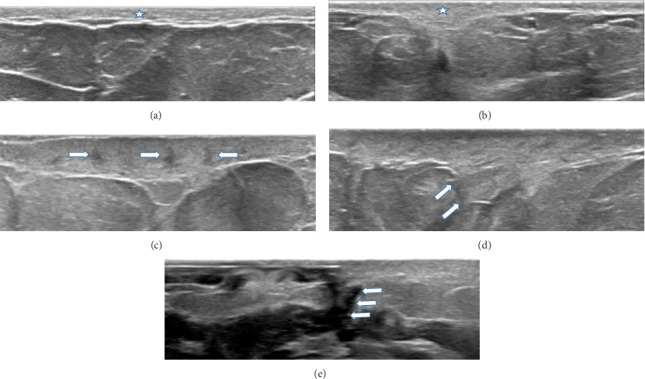
Echogenic fibrous bands extending linearly within the adipose tissue in normal breast skin (star) (a). Loss of regular linear echogenic fibrous structures within the adipose tissue and transformation into irregular, coarse echogenicity (star) (b). Small vacuoles formed in the thickened skin in later stages (arrows) (c). In the next stage, the echogenic linear line separating the skin and subcutaneous tissue is disrupted and the subcutaneous tissue is involved (arrows) (d). Formation of fistula tracts in the skin and subcutaneous tissue in the final stage (arrows) (e).

**Figure 2:**
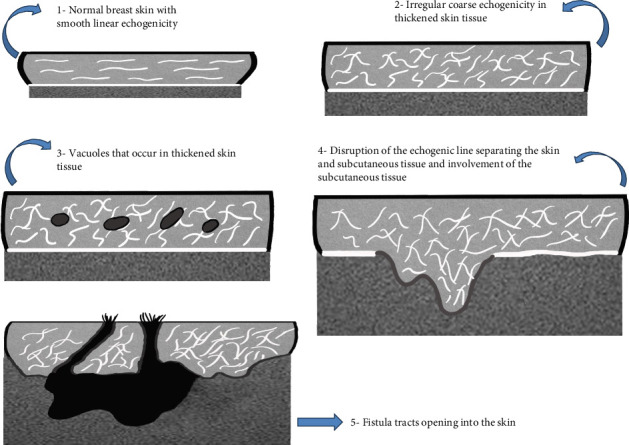
Sonographic schematic drawing of skin lesion development in IGM.

When stratified by symptom duration, the sonographic features demonstrated a sequential distribution. During the first 3 weeks, loss of fibrous echogenicity was the predominant abnormality (11/14, 78.6%), whereas vacuolar structures (2/14, 14.3%), dermo-subcutaneous blurring (1/14, 7.1%), and fistula formation (1/14, 7.1%) were uncommon. In the 4–6-week interval, vacuolar structures became more prevalent (11/19, 57.9%), and fistula formation was also observed (3/19, 15.8%). By 7–9 weeks, dermo-subcutaneous blurring (9/34, 26.5%) and fistula formation (4/34, 11.8%) were increasingly noted, in addition to persistent fibrous echogenicity loss (29/34, 85.3%). In patients with symptoms lasting ≥ 10 weeks, advanced cutaneous involvement was evident, most prominently characterized by fistula formation (11/17, 64.7%) and dermo-subcutaneous disruption (11/17, 64.7%). These sequential patterns are summarized in Table 1 and illustrated in Figure 1, with a schematic depiction provided in Figure 2.

### 3.2. Dermatological Findings

Clinical manifestations also exhibited a temporal distribution according to symptom duration. Within the first 3 weeks, erythema, papulopustular lesions, or cellulitis were the predominant findings (13/14, 92.9%), whereas erythema nodosum (1/14, 7.1%), ulceration (2/14, 14.3%), and fistula (1/14, 7.1%) were infrequent. At 4–6 weeks, erythema nodosum became more common (7/19, 36.8%), while ulceration (4/19, 21.1%) and fistula formation (3/19, 15.8%) also emerged. In the 7–9-week group, ulceration represented the most frequent clinical feature (16/34, 47.1%), often accompanied by erythema nodosum (5/34, 14.7%) and fistula formation (4/34, 11.8%). Among patients with symptom duration of ≥ 10 weeks, advanced cutaneous disease was dominated by chronic ulceration and fistula formation (11/17, 64.7%), whereas erythema and papulopustular lesions were rarely encountered (1/17, 5.9%). These temporal patterns are summarized in Table 2.

**Figure 3:**
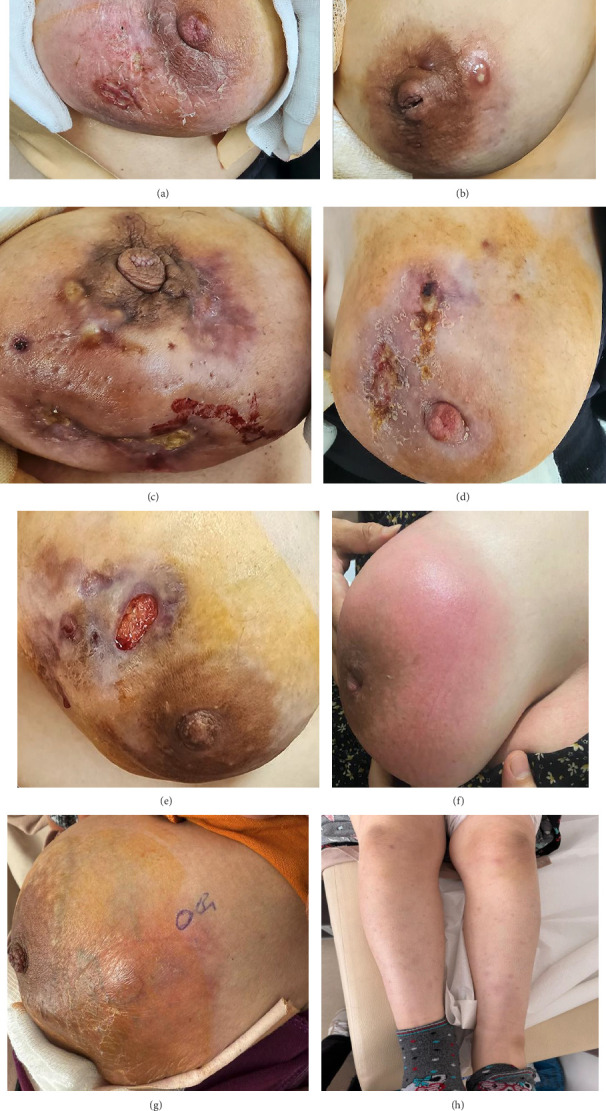
Ulcerated lesions in the lower inner quadrant of the left breast (a). Papular lesion with pustular center and surrounding erythema in the lower inner quadrant of the right breast (b). Painful ulcerated lesions with a purulent base and several pustular lesions around the areola in the lower inner quadrant and outer quadrant of the left breast (c). Painful ulcerated lesion with purulent discharge in places in the upper inner quadrant of the left breast (d). Oval ulcerated lesions in the upper outer quadrant of the right breast (e). Cellulitis in a patient with complaints of edema, pain, and redness in the upper quadrant of the right breast that started 4 weeks ago (f). The patient, who has complaints of edema, redness, and pain in the left breast that started 6–7 weeks ago and gradually increased, has had purplish plaque lesions on bilateral legs for the last 3 weeks. First, erythema nodosum was considered (g). Erythema nodosum plaques on the legs of the same patient (h).

Among the 14 patients who underwent skin biopsy, clinical dermatological manifestations were diverse. Ulcerated lesions were observed in 6 patients, while 2 patients exhibited papulopustular lesions. Erythema was noted in 2 patients, and erythema nodosum was detected in another 2. Additionally, cellulitis was documented in 2 cases, defined as a clinical diagnosis based on physical examination findings including erythema, edema, tenderness, and warmth of the skin (Figure 3).

### 3.3. Histopathological Findings

Breast tissue samples demonstrated classic features of lobulocentric GM, including granulomas composed of histiocytes, multinucleated giant cells, neutrophils, and lymphocytes. Although the histological findings were not pathognomonic, the differential diagnosis included IGM, cystic neutrophilic GM (often associated with *Corynebacterium*), sarcoidosis, infectious etiologies, and foreign body reactions. The absence of identifiable microorganisms, the lobulocentric pattern of inflammation, and negative results for specific stains (acid-fast bacilli, bacteria, and fungi) supported the diagnosis of IGM. No evidence of malignancy or foreign material was found.

**Figure 4:**
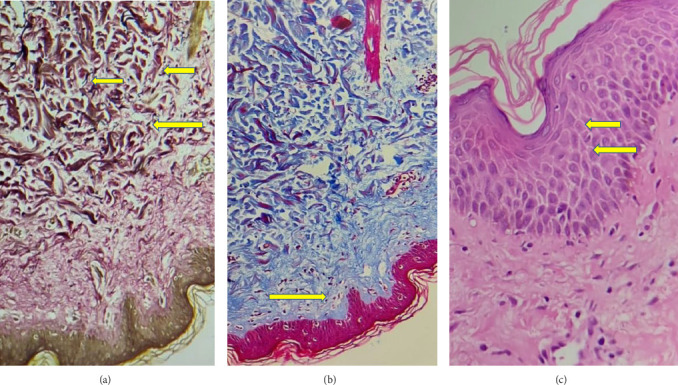
(Arrows) Elastin fiber degeneration (a). Papillary dermal fibrosis (b). Spongiosis (c).

**Figure 5:**
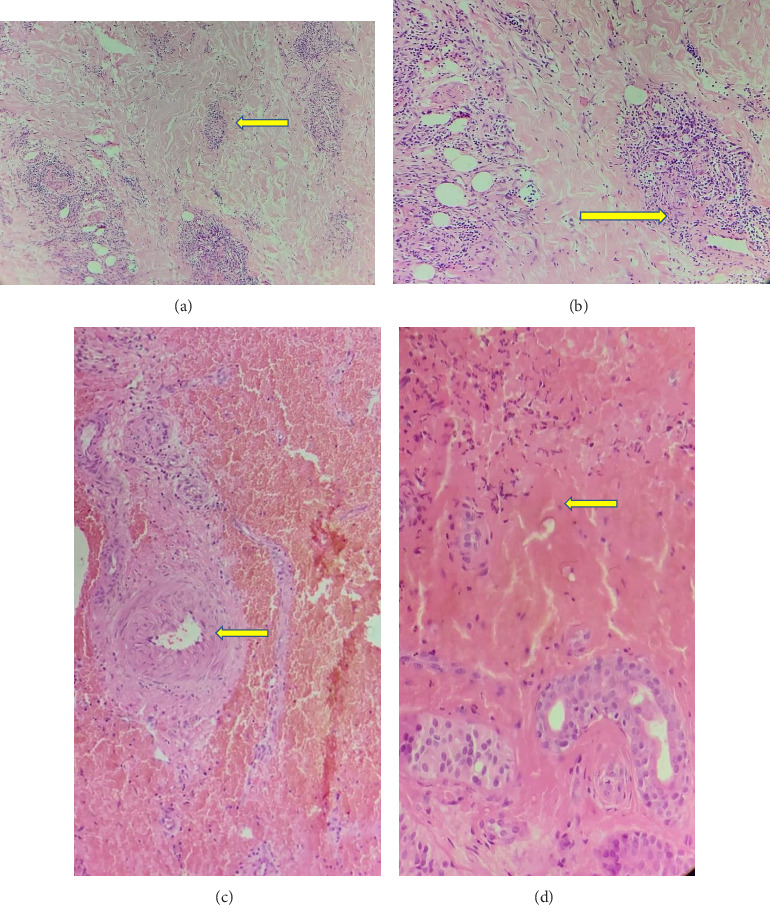
(Arrows) Full thickness dermal inflammation (a). Perivascular painful inflammation (b). Medium-sized vascular structure that vasculitis was not observed (c). Cell fragments within bleeding areas (d).

Among the 9 skin biopsy specimens available for detailed evaluation, spongiosis was observed in all cases (severe in one), while mild lymphocyte exocytosis was seen in 7 cases. Hyperkeratosis (parakeratosis) and epidermal acanthosis were each identified in one patient. All samples exhibited mild-to-moderate papillary dermal edema. Papillary dermal fibrosis was present in 3 cases, while damage to dermal elastin fibers was noted in 6 samples to varying degrees. Dermal perivascular inflammation was predominantly lymphocytic in 6 cases and neutrophilic in 2. Additionally, multinucleated giant cells were detected in septal regions. Notably, granuloma formation, vasculitis, vascular ectasia, or thrombus was not observed in any skin tissue (Figures 4 and 5).

## 4. Discussion

While the parenchymal involvement of IGM has been well-documented in multiple radiological and clinical studies [2, 4, 6], its skin manifestations and corresponding sonographic features remain notably underreported in the literature [11, 12].

Our most significant contribution lies in the identification of four novel sonographic findings associated with skin involvement in IGM, each of which appears to reflect distinct pathophysiologic processes. The first observed alteration was the loss of the regular horizontal fibrous echogenicity within the subcutaneous adipose tissue. This pattern likely reflects early inflammatory infiltration and stromal edema, leading to disorganization of collagen and elastin fibers, which are normally aligned in a parallel fashion [15].

The second finding—irregular and coarse echogenic lines—may represent ongoing granulomatous inflammation accompanied by fibroblastic proliferation and early fibrosis. As the immune response intensifies, the granulomatous aggregates disrupt the normal linear pattern and induce architectural distortion, which manifests sonographically as coarse echogenicity [19].

The third notable feature was the appearance of small vacuolar structures within the skin layers. These are hypothesized to be related to localized tissue necrosis, liquefactive degeneration, or microabscess formation within the inflamed dermis and subdermal tissue. Such changes are consistent with histological findings of spongiosis, edema, and focal dermal damage observed in our biopsy specimens [4].

The fourth and most advanced finding was the disruption of the hyperechoic boundary between the dermis and subcutaneous fat, indicating progression of inflammation beyond the dermal layers. The formation of fistulous tracts observed in this phase reflects a chronic inflammatory response with dermal remodeling and tissue breakdown. This progression parallels clinical findings of ulceration and purulent discharge in advanced cases of IGM [20].

The dermatologic examination findings—including erythema nodosum, papulopustular eruptions, and cellulitis-like presentations—highlight the spectrum of immune-mediated cutaneous responses in IGM. These manifestations, together with the sonographic progression described above, suggest that skin involvement in IGM may not merely be an extension of parenchymal inflammation but rather an active immunologic component of the disease.

The temporal distribution of clinical manifestations (Table 2) paralleled the sonographic findings (Table 1). Early cases were dominated by erythema and papulopustular changes, whereas ulceration and fistula formation emerged in patients with longer disease duration. This consistency between clinical and imaging features reinforces the concept of a sequential progression, although definitive temporal staging remains limited by patient-reported data.

Histopathological analysis of skin biopsies further supports this concept. The absence of granulomas in skin tissue, despite their presence in breast parenchyma, suggests that dermal involvement may be mediated through a different inflammatory mechanism, possibly resembling a delayed-type hypersensitivity reaction or secondary immunologic response rather than a primary granulomatous process [21].

In conclusion, the novel sonographic features identified in this study provide valuable insight into the cutaneous dynamics of IGM and may serve as supportive clinical clues in patients with suspected IGM. Understanding the sequential dermal changes and their underlying pathophysiologic mechanisms can guide clinicians in diagnosis, monitoring, and tailoring targeted therapies for patients with extensive cutaneous disease. However, they should not be considered sufficient for diagnosis in isolation, and histopathological confirmation remains mandatory.

## 5. Conclusion

This study provides a novel perspective on the cutaneous manifestations of IGM, an aspect that has been underrepresented in the radiology literature. Through detailed sonographic analysis, we identified a stepwise progression of skin involvement characterized by four novel ultrasound findings, each reflecting underlying pathophysiological changes. These observations—ranging from disruption of fibrous echogenicity to fistula formation—demonstrate that skin involvement in IGM is a dynamic and diagnostically valuable component of the disease. Histopathological evaluation further supported the presence of dermal inflammation, reinforcing the hypothesis that cutaneous involvement in IGM may represent a distinct immunologic process rather than simple extension of parenchymal disease. Awareness of these findings may improve diagnostic confidence, guide biopsy decisions, and potentially inform earlier intervention strategies.

### 5.1. Limitations

This study has several limitations. First, it was a single-center analysis, which may limit the generalizability of the findings. Second, although 14 patients underwent skin biopsy, histopathologic sampling may not have fully captured the entire spectrum of cutaneous involvement, and granulomatous features were not observed in all cases. Third, microbiological correlation was limited to conventional staining methods; advanced molecular techniques such as PCR or 16S rRNA sequencing were not employed to exclude rare infectious etiologies. Finally, follow-up imaging after steroid treatment was not standardized for all patients, making it difficult to evaluate sonographic resolution or progression consistently.

## Figures and Tables

**Table 1:** Distribution of sonographic findings according to symptom duration in patients with idiopathic granulomatous mastitis.

The time between the patient's first complaint and admission	Number of patients (*n*)	Fibrous echogenicity loss (*n*)	Vacuolar structures (*n*)	Dermo-subcutaneous blurring (*n*)	Fistula formation (*n*)
0–3 weeks	14	11	2	1	1
4–6 weeks	19	14	11	2	3
7–9 weeks	34	29	15	9	4
≥ 10 weeks	17	17	14	11	11

**Table 2:** Distribution of clinical cutaneous findings according to symptom duration in patients with idiopathic granulomatous mastitis.

The time between the patient's first complaint and admission	Number of patients (*n*)	Erythema + papulopustule + cellulitis (*n*)	Erythema nodosum (*n*)	Ulcer (*n*)	Chronic ulcers and fistulas (*n*)
0–3 weeks	14	13	1	2	1
4–6 weeks	19	12	7	4	3
7–9 weeks	34	7	5	16	4
≥ 10 weeks	17	1	1	6	11

## Data Availability

The data that support the findings of this study are available from the corresponding author upon reasonable request.

## Nomenclature

CDC: Centers for Disease Control and Prevention
GM: Granulomatous mastitis
HLA: Human leukocyte antigen
IGM: Idiopathic granulomatous mastitis
TB: Tuberculosis
